# ﻿Reassessment of the type locality of *Euptychiastigmatica* Godman, 1905, with the description of two new sibling species from Amazonia (Lepidoptera, Nymphalidae, Satyrinae, Satyrini)

**DOI:** 10.3897/zookeys.1167.102979

**Published:** 2023-06-14

**Authors:** Shinichi Nakahara, Kaylin Kleckner, Eduardo P. Barbosa, Giselle M. Lourenço, Mirna M. Casagrande, Keith R. Willmott, André V. L. Freitas

**Affiliations:** 1 Department of Organismic and Evolutionary Biology, Museum of Comparative Zoology, Harvard University, 26 Oxford Street, Cambridge, MA 02138, USA; 2 Honey Bee Research and Extension Laboratory, Entomology and Nematology Department, University of Florida, Gainesville, FL 32611, USA; 3 Departamento de Biologia Animal and Museu de Diversidade Biológica, Instituto de Biologia, Universidade Estadual de Campinas – UNICAMP. 13083-970 Campinas, São Paulo, Brazil; 4 Departamento de Biologia Geral, Instituto de Ciências Biológicas, Universidade Federal de Minas Gerais – UFMG. 31270-901 Belo Horizonte, Minas Gerais, Brazil; 5 Departamento de Zoologia, Universidade Federal do Paraná, Curitiba, Paraná, Brazil; 6 McGuire Center for Lepidoptera and Biodiversity, Florida Museum of Natural History, University of Florida, Gainesville, FL 32611, USA

**Keywords:** Atlantic Forest, Brazil, Entre Rios, Euptychiina, Herbert Huntingdon Smith, Peru, Rio de Janeiro, taxonomy

## Abstract

A brief historical review regarding the type locality of *Euptychiastigmatica* Godman, 1905 was conducted, which suggests that its type locality is actually Rio de Janeiro, Brazil, rather than northeastern Argentina, as previously purported. Consequently, *E.stigmatica* and its senior synonym *E.cyanites* Butler, 1871, are regarded to be two species-group names representing a taxon in the euptychiine genus *Caeruleuptychia* Forster, 1964 known from the Brazilian Atlantic Forest. A lectotype is designated for *E.cyanites*. Additionally, two closely related species are named and described using an integrative approach with morphological and molecular evidence. *Caeruleuptychiaharrisi* Nakahara & Freitas, **sp. nov.** and *C.aemulatio* Nakahara & Willmott, **sp. nov.** both occur in Amazonia and COI barcode data recovered these taxa as part of the *caerulea* clade of *Caeruleuptychia*.

## ﻿Introduction

Amazonia and the Atlantic Forest are two of the four major biogeographical regions in the Neotropical realm. Despite the existence of several definitions of the term “Amazon” (e.g., Eva et al. 2005), it hardly needs saying that this word applies to the largest contiguous region of tropical rainforests on earth, situated in South America encompassing nine countries: Bolivia, Brazil, Colombia, Ecuador, French Guiana, Guyana, Peru, Suriname, and Venezuela. The highly diverse but vanishing Atlantic Forest spans more than 3,000 km along the southeastern coast of Brazil and penetrates into the northern edge of Argentina and southeastern Paraguay (Morellato and Haddad 2000; Giraudo 2003; Huang et al. 2007; Ribeiro et al. 2009). These two biogeographical regions are currently separated from each other by a diagonal series of open vegetation formations encompassing the semi-arid Caatinga, the Cerrado savannas, and the dry Chaco (Santos et al. 2012; Bonatelli et al. 2022). Nevertheless, mounting evidence supports historical biotic connections between the Amazon and the Atlantic Forest (e.g., Brown 1987; Costa 2003; Pavan et al. 2011; Batalha-Filho et al. 2013; Rodrigues et al. 2014), and many sister species pairs are known to have their distribution restricted to either one of these two major biogeographical regions (e.g., Silva et al. 2014). In addition, faunistic and floristic similarities have been reported in certain parts of these biogeographical regions: the “tabuleiro” forests (= tableland forests) in the Brazilian states of Espírito Santo and southern Bahia show floristic resemblance with Amazonia by sharing 223 plant species (Andrade-Lima 1966), and faunistic similarity between these two biogeographical regions has also been indicated (Coelho et al. 2022). Recent discoveries of new species from the tableland forests of Espírito Santo further strengthen the affinity of this region with Amazonia (e.g., Lewis et al. 2017).

The butterfly subtribe Euptychiina is a diverse radiation in the Neotropics, and our understanding of its systematics has seen steady improvement (e.g., Espeland et al. 2023). As members of one of the more diverse euptychiine genera, *Caeruleuptychia* Forster, 1964 species are well represented throughout the lowlands of tropical South America east of the Andes. Ongoing work using both morphological and molecular data recognizes three clades within *Caeruleuptychia*: 1) a group with species exhibiting sexual dimorphism and males displaying an androconial patch on the dorsal hindwing (*aegrota* clade); 2) a group mainly including species with blue-lilac wings and possessing a concavity along the dorsal margin of the male genitalic valvae (*caerulea* clade); 3) a group including mainly species with brown wings, with males accompanied by secondary sexual structures (*umbrosa* clade). There exist a number of other morphological characters to support each of these three clades (e.g., Nakahara et al. 2020a), and a study is underway to revise the latter two clades. Nevertheless, the *aegrota* clade has recently been revised and its known species diversity may not increase (Nakahara et al. 2018b). On the other hand, our understanding of species richness in the *caerulea* clade and *umbrosa* clade is far from complete, since COI barcoding data suggest they contain a high number of cryptic species (unpublished data), which otherwise might have remained unrecognized based solely on external morphology. Given this situation, cataloging *Caeruleuptychia* diversity in the form of single to few species descriptions provides progress towards developing a firm species-level classification of the group. The urgency to document *Caeruleutychia* species richness in Amazonia and the Atlantic Forest biomes is increased by the high degrees of biodiversity loss in these regions, driven by forest loss, fragmentation, and degradation (e.g., de Lima et al. 2020; da Cruz et al. 2021).

Therefore, we here propose taxonomic changes in the *caerulea* clade of *Caeruleuptychia* in order to contribute towards cataloging species-level diversity of the genus in these distinctive biogeographical regions. In the current study, we discuss the taxonomic status and history of two species-group names currently placed in *Caeruleuptychia*, *Euptychiacynanites* Butler, 1871 and *E.stigmatica* Godman, 1905. These two species-group names were considered synonymous by Weymer (1911) and the identity of these two names has not been discussed by any subsequent authors with evidence. Here, we reinterpret the type locality of *E.stigmatica* and regard this nomen as a species-group name to represent a *Caeruleuptychia* taxon known from the Atlantic Forest. Further, we provide evidence to retain *E.stigmatica* as a junior subjective synonym of *E.cyanites*. Additionally, we name and describe two closely related undescribed Amazonian *Caeruleuptychia* species utilizing an integrative approach.

## ﻿Materials and methods

### ﻿Morphological work and acronyms

We studied external morphological characters using a Leica MZ 16 stereomicroscope, with camera lucida attached, at the FLMNH, as well as a Zeiss SteREO Discovery V.20 Stereomicroscope in association with the AxioVision Rel.4.8 software for focus-stacking images at the ZUEC. The genitalia were examined by separating the abdomen from the body and soaking in hot 10% potassium hydroxide solution (KOH) for a few minutes. Voucher information for these dissected specimens is provided below. Terminology associated with wings (area, venation, elements etc.) and genitalia follow Nakahara et al. (2018a). The information on male individuals used to record forewing length and forewing scent patch size are tabulated in Table 1. We visualized these measurements and created bivariate scatterplots using the ggplot2 package (Wickham 2016) in R v. 4.1.3 to visually inform our taxonomic hypotheses discussed herein.

**Table 1.** Measurements of forewing (FW) length and FW scent patch size (i.e., length of FW scent patch size proportional to the inner margin length (%)) used to produce bivariate scatterplot shown in Fig. 1B.

Voucher	Species	Repository	Locality	Fw length (Mm)	Scent patch size(%)
BLU778	*Caeruleuptychiacyanites*	ZUEC	Minas Gerais, Brazil	25	25
ZUEC-LEP 12054	*Caeruleuptychiacyanites*	ZUEC	Minas Gerais, Brazil	26,1	31
ZUEC-LEP 12055	*Caeruleuptychiacyanites*	ZUEC	Minas Gerais, Brazil	25	26
ZUEC-LEP 12056	*Caeruleuptychiacyanites*	ZUEC	Minas Gerais, Brazil	26	29
ZUEC-LEP 12057	*Caeruleuptychiacyanites*	ZUEC	Minas Gerais, Brazil	25	28
ZUEC-LEP 12058	*Caeruleuptychiacyanites*	ZUEC	Minas Gerais, Brazil	25,2	29
ZUEC-LEP 12059	*Caeruleuptychiacyanites*	ZUEC	Minas Gerais, Brazil	26,1	29
FLMNH 1138898	*Caeruleuptychiacyanites*	FLMNH	Espírito Santo, Brazil	25	27
N/A	*Caeruleuptychiacyanites*	DZUP	Minas Gerais, Brazil	24,4	29
**BMNH (E) 1267020**	***Caeruleuptychiacyanites* (*stigmatica* holotype)**	**NHMUK**	**Rio de Janeiro, Brazil**	**25,4**	**27**
**BMNH (E) 1267021**	***Caeruleuptychiacyanites* (*cyanites* lectotype)**	**NHMUK**	**Brazil**	**25**	**28**
N/A	*Caeruleuptychiacyanites*	MfN	“Sao Paulo”, Brazil	23	27
N/A	*Caeruleuptychiacyanites*	MfN	Casabranca, Brazil	23,5	26
DZ 40.523	*Caeruleuptychiacyanites*	DZUP	Espírito Santo, Brazil	23,1	30
DZ 40.372	*Caeruleuptychiacyanites*	DZUP	Espírito Santo, Brazil	22,8	29
BMNH (E) 1717835	*Caeruleuptychiacyanites*	NHMUK	“Rio R[eal]”	23,9	28
MGCL-LOAN 546	*Caeruleuptychiaharrisi*	ZUEC	Rondônia, Brazil	22,4	14
MGCL-LOAN 561	*Caeruleuptychiaharrisi*	ZUEC	Rondônia, Brazil	22,2	19
FLMNH 279345	*Caeruleuptychiaharrisi*	FLMNH	Rondônia, Brazil	21,1	15
**USNM ENT 01844280**	***Caeruleuptychiaharrisi* (holotype)**	**USNM**	**Rondônia, Brazil**	**20,2**	**17**
DZ 57.996	*Caeruleuptychiaharrisi*	DZUP	Rondônia, Brazil	21	16
MGCL-LOAN-447	*Caeruleuptychiaaemulatio*	ZUEC	Acre, Brazil	22,6	21
MGCL-LOAN-452	*Caeruleuptychiaaemulatio*	ZUEC	Acre, Brazil	21,7	21
FLMNH 1138899	*Caeruleuptychiaaemulatio*	FLMNH	Pasco, Peru	21,6	26
N/A	*Caeruleuptychiaaemulatio*	USNM	Cuzco, Peru	22	21
DZ 57.994	*Caeruleuptychiaaemulatio*	DZUP	Acre, Brazil	21	27
DZ 57.995	*Caeruleuptychiaaemulatio*	DZUP	Acre, Brazil	20	27
FLMNH 279343	*Caeruleuptychiaaemulatio*	FLMNH	Madre de Dios, Peru	20,8	23
MUSM-LEP 100090	*Caeruleuptychiaaemulatio*	MUSM	Madre de Dios, Peru	20,7	23
**MUSM-LEP 100094**	***Caeruleuptychiaaemulatio* (holotype)**	**MUSM**	**Madre de Dios, Peru**	**20,2**	**25**
MUSM-LEP 100095	*Caeruleuptychiaaemulatio*	MUSM	Madre de Dios, Peru	21,4	20

The following collections and acronyms are used throughout the text: **AMNH** – American Museum of Natural History, New York, USA; **DZUP** – Entomological Collection Pe. Jesus Santiago Moure, Universidade Federal do Paraná, Curitiba, Brazil; **FLMNH** – McGuire Center for Lepidoptera and Biodiversity, Florida Museum of Natural History, Gainesville, USA; **MfN** – Museum für Naturkunde, Leibniz-Institut für Evolutions-und Biodiversitätsforschung, Berlin, Germany; **MNRJ** – Museu Nacional, Universidade Federal do Rio de Janeiro, Rio de Janeiro, Brazil; **NHMUK** – The Natural History Museum, London, UK; **VOB** – Vitor O. Becker Collection, Camacan, Brazil; **USNM** – National Museum of Natural History, Smithsonian Institution, Washington, DC, USA; **ZUEC** – Zoological Collection of the Museu de Diversidade Biológica da Universidade Estadual de Campinas, Campinas, Brazil.

### ﻿Molecular work

We sequenced the cytochrome c oxidase I (COI) barcode region (sensu Hebert et al. 2003) for relevant *Caeruleuptychia* taxa to inform our taxonomic hypothesis. Genomic DNA extraction methods, PCR conditions and various primers used to amplify this gene largely follow Nakahara et al. (2020b). New sequences are uploaded to GenBank and information for all sequences used in the present study is tabulated in Table 2. The dataset comprised a total of 55 individuals (54 individuals of selected *Caeruleuptychia* taxa and *Capronnieriagalesus* (Godart, [1824])) of 630 base pairs edited in various versions of Geneious (Biomatters Ltd.; Kearse et al. 2012). A Maximum-likelihood (ML) tree was inferred in IQ-TREE v. 2.1.0 (Minh et al. 2020), with the best-fit substitution models for each codon position through ModelFinder (-m MFP) (Kalyaanamoorthy et al. 2017) (1^st^ codon position: TNe+FQ+R2; 2^nd^ codon position: HKY+F; 3^rd^ codon position: TIM+F+G4). We conducted ten independent likelihood searches under these models, and the tree with the highest log-likelihood (LnL=-2678.083) was selected and *Capronnieriagalesus* was used to root the tree. Branch support was calculated using 1,000 replications of ultrafast bootstrap (UFBoot) (Hoang et al. 2018), optimized with nearest neighbor interchange and 1,000 replicates of SH-like approximate Likelihood Ratio Test (SH-aLRT) (Guindon et al. 2010). IQ-TREE analysis was run on the FASRC Cannon cluster supported by the FAS Division of Science Research Computing Group at Harvard University (Cambridge, USA). The above dataset was also used to calculate pairwise distances based on Kimura 2-parameter distance model in MEGA 11 (Tamura et al. 2021).

**Table 2.** GenBank voucher information for sequenced material in this work.

Voucher code	Species	Locality	GenBank ID
NW167-5	*Capronnieriagalesus*	Brazil: Santa Catarina	GU205826
BC-DZ-Willmott-004	*Caeruleuptychiaaetherialis*	Brazil: Acre: 9.2 km SE Santa Rosa do Purus	MF192688
BC-DZ-Willmott-017	*Caeruleuptychiaglauca*	Brazil: Acre: 6.4 km E Santa Rosa do Purus	MF192715
BC-DZ-Willmott-018	*Caeruleuptychiaglauca*	Brazil: Acre: 50 km NW Bujari	OP824987
KW-15-091	*Caeruleuptychiaglauca*	Peru: Cuzco: Quebrada Quitacalzón	MF192714
CP01-11	*Caeruleuptychiahelios*	Peru: Madre de Dios: Tambopata Research Center	GU205822
CP01-91	*Caeruleuptychiahelios*	Peru: Madre de Dios: Tambopata Research Center	DQ338584
CP02-43	*Caeruleuptychia* sp. nov.	Peru: Madre de Dios: Posada Amazonas	GU205825
KW-15-098	*Caeruleuptychia* sp. nov.	Peru: Madre de Dios: Albergue Amazonía	MF192710
KW-15-099	*Caeruleuptychia* sp. nov.	Peru: Cuzco: Villa Carmen	MF192711
LEP-18630	*Caeruleuptychia* sp. nov.	Peru: Cuzco: Quebrada Quitacalzón	OP824992
LEP-37192	*Caeruleuptychia* sp. nov.	Peru: Madre de Dios: Los Amigos Biological Station	OP824993
LEP-37212	*Caeruleuptychia* sp. nov.	Peru: Madre de Dios: Los Amigos Biological Station	OP824994
MGCL-LOAN-272	*Caeruleuptychia* sp. nov.	Brazil: Acre: Reserva Cazumbá-Iracema	MF192709
MGCL-LOAN-448	*Caeruleuptychia* sp. nov.	Brazil: Acre: Foz do Rio Tejo, estrada para o Rio Arara	MF192683
MGCL-LOAN-449	*Caeruleuptychia* sp. nov.	Brazil: Acre: Foz do Rio Breu	MF192708
MGCL-LOAN-543	*Caeruleuptychia* sp. nov.	Brazil: Rondônia: Abunã	MF192716
E-40-11	*Caeruleuptychiacoelica*	Not recorded	AY508524
LEP-09565	*Caeruleuptychiacoelica*	Ecuador: Morona-Santiago: Río Abanico	MF192697
LEP-09566	*Caeruleuptychiacoelica*	Ecuador: Morona-Santiago: Río Abanico	OP824989
KW-090113-23	*Caeruleuptychiaaegrota*	Ecuador: Orellana: Boca del Río Añangu	OP824988
LEP-37520	*Caeruleuptychiaaegrota*	Ecuador: Napo: Río Chalayacu	MF192686
LEP-14941	*Caeruleuptychiaaegrota*	Ecuador: Zamora-Chinchipe: hill above Quebrada Maycú	MF192687
KW-15-008	*Caeruleuptychiahelios*	Peru: Madre de Dios: Los Amigos Biological Station	MF192693
KW-15-009	*Caeruleuptychiahelios*	Peru: Madre de Dios: Albergue Amazonía	MF192694
KW-15-092	*Caeruleuptychiahelios*	Peru: Madre de Dios: Albergue Amazonía	MF192695
LCB260	*Caeruleuptychiahelios*	French Guiana: St-Laurent du Maroni: Saül	MF192719
MGCL-LOAN-441	*Caeruleuptychiahelios*	Brazil: Acre: Tabocal do Nonato, caminho para Rio Arara	MF192692
KW-15-088	*Caeruleuptychiadivina*	Peru: Cuzco: San Pedro	MF192696
LEP-08906	*Caeruleuptychiatrembathi*	Ecuador: Zamora-Chinchipe: km 18 Los Encuentros-Zarza	MF192690
LEP-15075	*Caeruleuptychiatrembathi*	Ecuador: Morona-Santiago: 2 km N San Isidro	MF192691
LEP-15076	*Caeruleuptychiatrembathi*	Ecuador: Morona-Santiago: 2 km N San Isidro	MF192689
LEP-15077	*Caeruleuptychiatrembathi*	Ecuador: Morona-Santiago: 2 km N San Isidro	OP824991
LEP-15175	*Caeruleuptychiatrembathi*	Ecuador: Morona-Santiago: Río Abanico	OP824990
KW-15-100	*Caeruleuptychiaurania*	Peru: Cuzco: Chontachaca	MF192706
KW-15-101	*Caeruleuptychiaurania*	Peru: Madre de Dios: Albergue Amazonía	MF192684
KW-15-104	*Caeruleuptychiaurania*	Peru: Madre de Dios: Albergue Amazonía	MF192705
KW-15-105	*Caeruleuptychiaurania*	Peru: Madre de Dios: Albergue Amazonía	MF192707
MGCL-LOAN-294	*Caeruleuptychiaurania*	Brazil: Mato Grosso: Cachoeira Sete Quedas	OP824995
MGCL-LOAN-547	*Caeruleuptychiaurania*	Brazil: Rondônia: Caiçara	MF192701
MGCL-LOAN-554	*Caeruleuptychiaurania*	Brazil: Rondônia: Caiçara	MF192703
MUSM-SN-17-1	*Caeruleuptychiaurania*	Peru: San Martín: La Unión	OP824996
LEPAR448-11	*Caeruleuptychiahelena*	Argentina: Misiones: Departamento de Iguazú: Parque Nacional Iguazú: Seccional Yacui	MF546867
LEPIG303-11	*Caeruleuptychiahelena*	Argentina: Misiones: Departamento de Iguazú: Parque Nacional Iguazú: Seccional Timbó	MF546098
MGCL-LOAN-351	*Caeruleuptychiapilata*	Brazil: Acre: Foz do Rio Tejo	OP824997
**MGCL-LOAN-447**	***Caeruleuptychiaaemulatio***	**Brazil: Acre: Foz do Rio Tejo, estrada para o Rio Arara**	**MF192712**
**MGCL-LOAN-452**	***Caeruleuptychiaaemulatio***	**Brazil: Acre: Foz do Rio Breu**	**MF192713**
**MGCL-LOAN-546**	***Caeruleuptychiaharrisi***	**Brazil: Rondônia: Caiçara**	**MF192717**
**MGCL-LOAN-561**	***Caeruleuptychiaharrisi***	**Brazil: Rondônia: Caiçara**	**MF192681**
**BC-DZ-Willmott-007**	***Caeruleuptychiacyanites***	**Brazil: Espírito Santo: Res. Ecológica Sooretama**	**OP824982**
**BLU778**	***Caeruleuptychiacyanites***	**Brazil: Minas Gerais: Lagoa Bonita: Parque Estadual do Rio Doce**	**MF192718**
**YPH0852**	***Caeruleuptychiacyanites***	**Brazil: Minas Gerais: Timóteo: Parque Estadual do Rio Doce**	**OP824983**
**YPH0853**	***Caeruleuptychiacyanites***	**Brazil: Minas Gerais: Timóteo: Parque Estadual do Rio Doce**	**OP824984**
**YPH0854**	***Caeruleuptychiacyanites***	**Brazil: Minas Gerais: Timóteo: Parque Estadual do Rio Doce**	**OP824985**
**LBR0654**	***Caeruleuptychiacyanites***	**Brazil: Espírito Santo: Sooretama: Parque Estadual de Sooretama**	**OP824986**

### ﻿Adult ecological study

Adults of *C.cyanites* were studied through a 12-month trap study in Rio Doce State Park (hereafter PERD, following the Portuguese acronym) (19°48'–19°0'S and 42°38'–42°28'W), in the municipalities of Marliéria, Timóteo, and Dionísio, state of Minas Gerais, Brazil. Ninety traps were placed in nine transects at two heights: 45 traps in the understory (1.5 m above ground) and 45 traps in the canopy (5–15 m above ground, beneath tree crowns). Traps were installed in groups of ten per transect at alternating heights to avoid the interference of canopy traps on understory traps. A standard mixture of mashed banana with sugar cane juice, fermented for at least 48 h, was used as an attractant. The bait was placed in plastic pots with a perforated cover inside the traps, which were checked every 48 h and were replaced at each visit. All traps were kept open simultaneously in the field for consecutive periods of five days. Butterflies were sampled monthly from August 2015 to July 2016. All butterflies were marked with an individual number on the ventral surface of the hind wings and released unharmed. For further details about the study site and methods, see Lourenço et al. (2019).

## ﻿Results

### ﻿ Caeruleuptychia cyanites

Taxon classificationAnimaliaLepidopteraNymphalidae

(Butler, 1871)

4DCCB9E7-19DC-5CA1-8465-16D5CE03A32A

**Figure 1.**
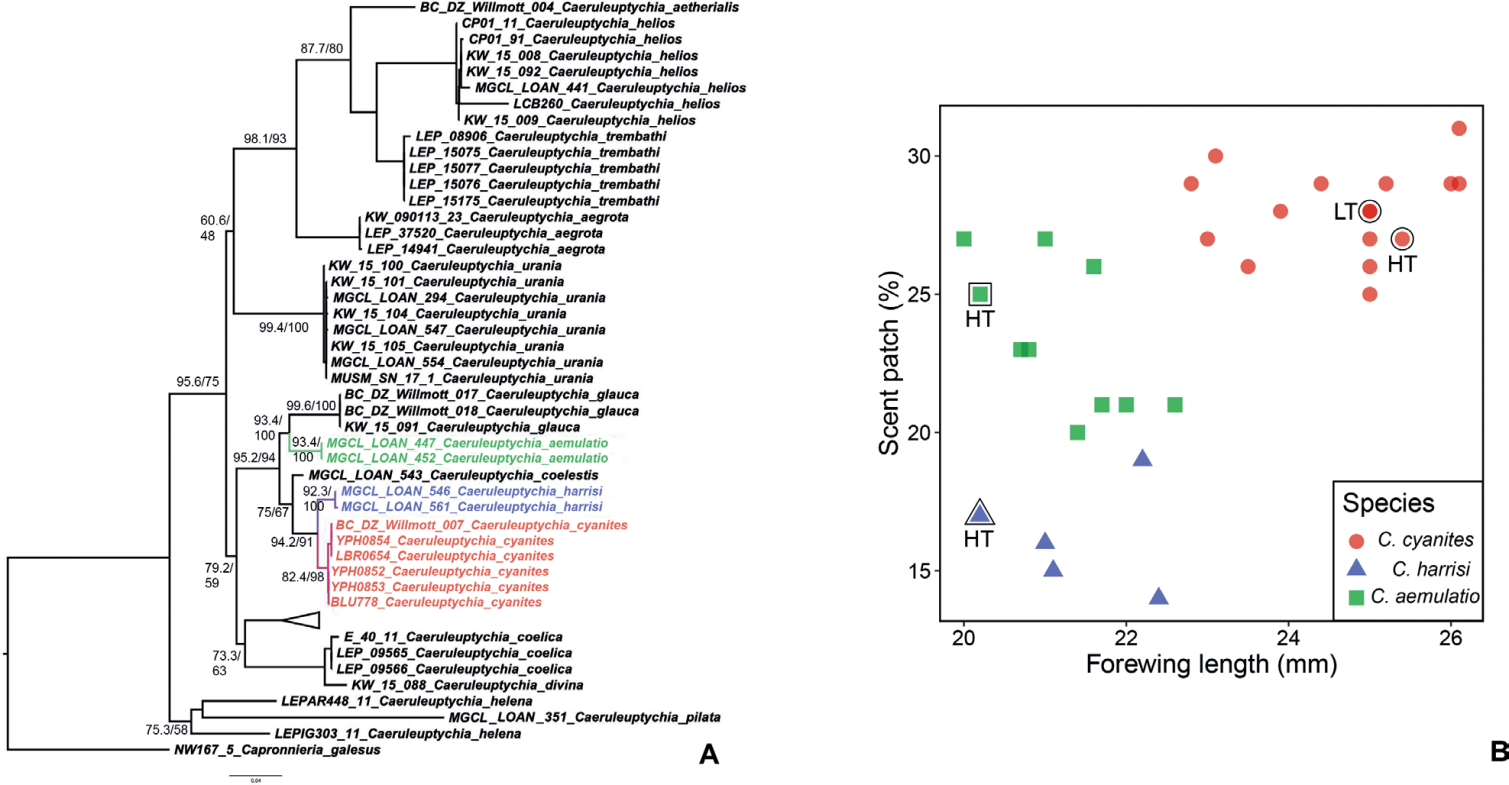
**A** maximum likelihood tree (LnL = -2678.083) showing the phylogenetic relationships among *Caeruleuptychia* taxa selected for this study. Numbers beside branches are UFBoot/SH-aLRT values; colored tips represent taxonomic hypotheses proposed in the present study **B** the differentiation of three *Caeruleuptychia* species (*C.cyanites*, *C.harrisi* sp. nov., *C.aemulatio* sp. nov.) by forewing length (mm) and scent patch percentage. Each species is denoted by a unique color and shape with a single point representing a single specimen. The holotype (HT) of each name and the lectotype (LT) of *C.cyanites* are denoted by a black border and the corresponding letters.

**Figure 2.**
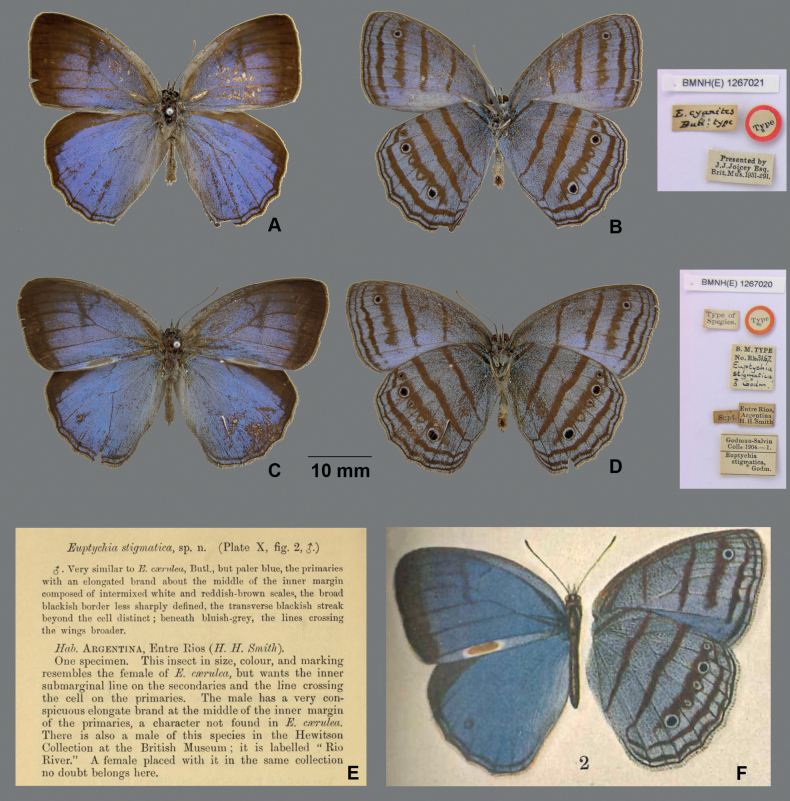
Lectotype of *E.cyanites* (NHMUK) **A** in dorsal view **B** in ventral view, with associated labels to the right. Holotype of *E.stigmatica* (NHMUK) and its original description **C** in dorsal view **D** in ventral view, with associated labels to the right **E** text of the original description by Godman (1905: 186) **F** illustration associated with Godman (1905: pl. 10, fig. 2).

**Figure 3.**
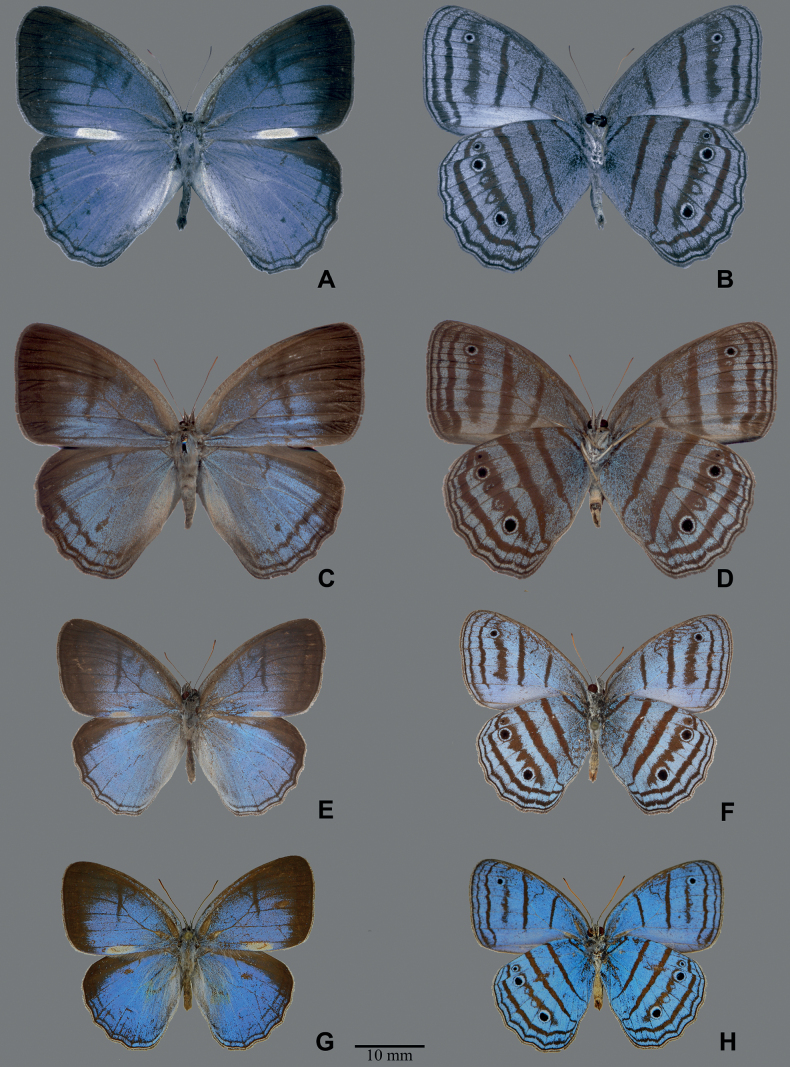
Adults of *Caeruleuptychia*: *C.cyanites***A** male in dorsal view **B** male in ventral view (BLU778) **C** female in dorsal view **D** female in ventral view (BC-DZ-007). Holotype male of *Caeruleuptychiaharrisi* sp. nov. **E** dorsal view **F** ventral view (USNM ENT 01844280). Holotype male of *Caeruleuptychiaaemulatio* sp. nov. **G** dorsal view **H** ventral view (MUSM-LEP 100094).

**Figure 4.**
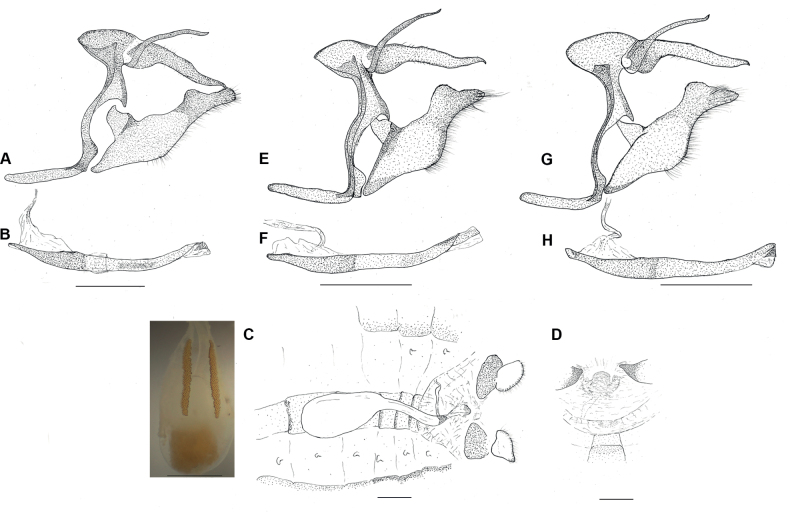
Genitalia of *C.cyanites***A** male genitalia in lateral view, without phallus **B** phallus in lateral view (genitalic dissection: SN-19-13) **C** female genitalia in dorsal view, with image of signa to the left **D** lamella antevaginalis in ventral view (genitalic dissection: SN-19-14). *Caeruleuptychiaharrisi* sp. nov. **E** male genitalia in lateral view, without phallus **F** phallus in lateral view (genitalic dissection: SN-17-36); *Caeruleuptychiaaemulatio* sp. nov. **G** male genitalia in lateral view, without phallus **H** phallus in lateral view (genitalic dissection: SN-17-34). Scale bars: 1 mm.

**Figure 5.**
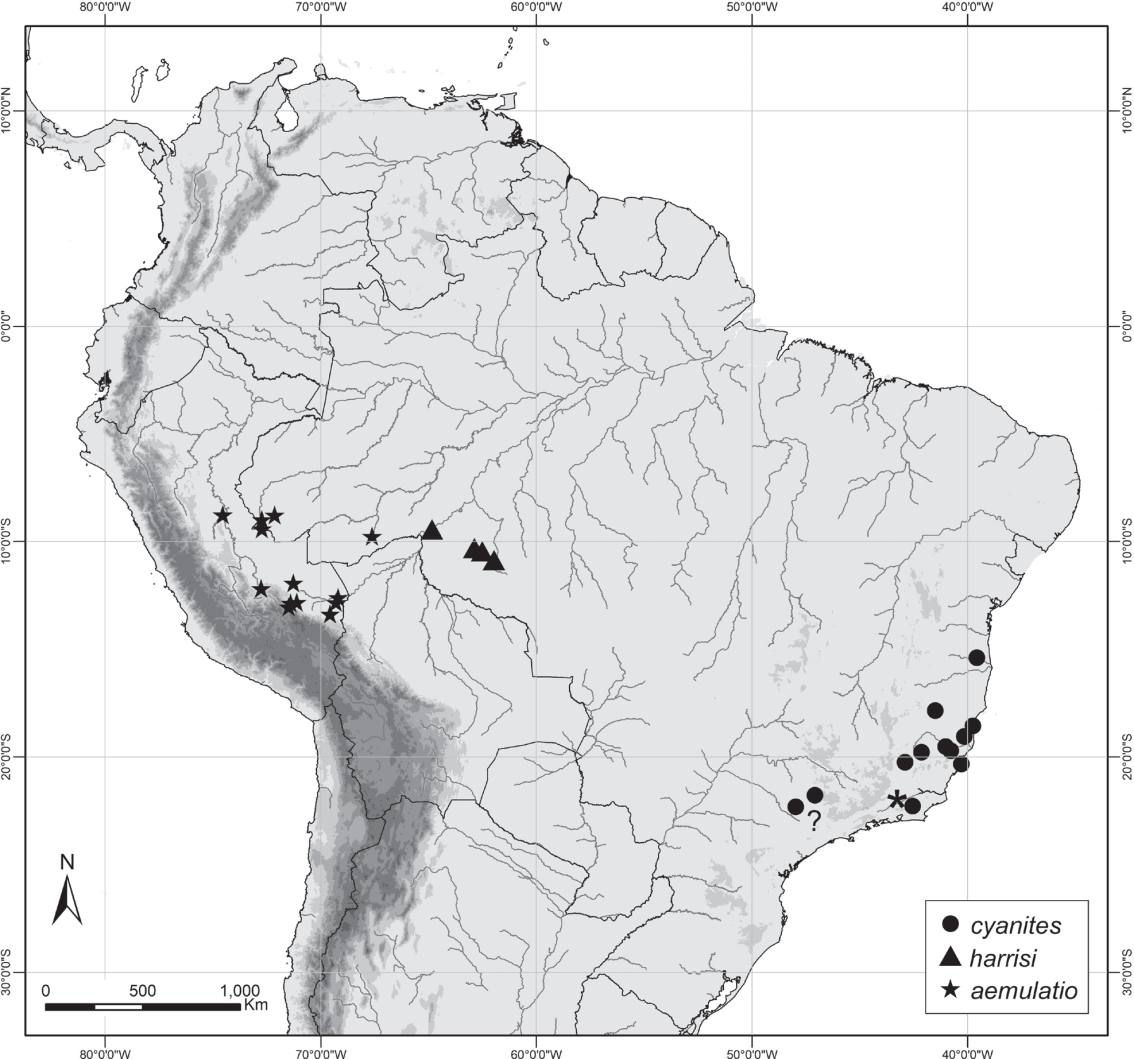
Distribution map for three *Caeruleuptychia* species discussed herein. Key: * denotes location of Três Rios, Rio de Janeiro (= hypothesized type locality of *E.stigmatica* in the present study); ? denotes dubious records from Araras, São Paulo.

**Figure 6.**
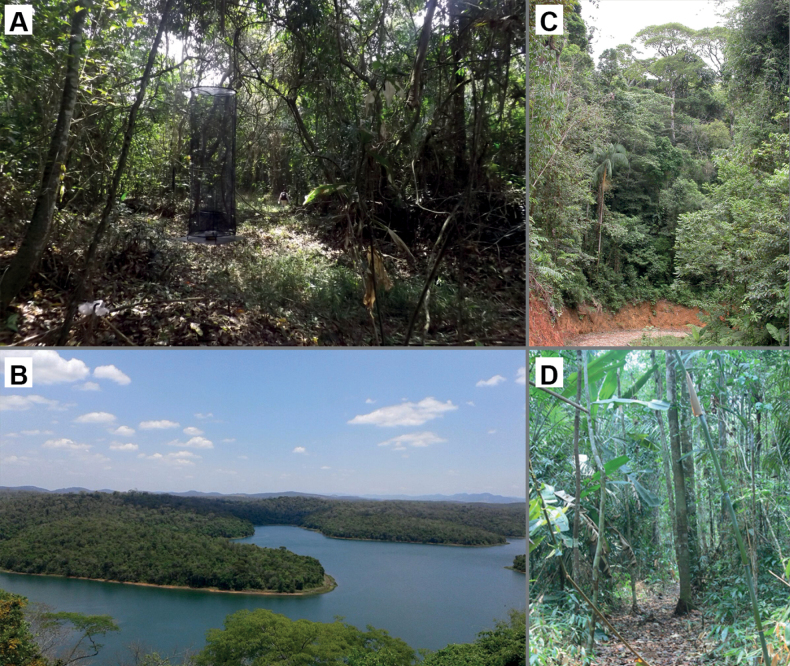
Habitat pictures for two *Caeruleuptychia* species discussed herein **A** interior of ecotone habitat of *C.cyanites* at PERD with trap pictured **B** general view of PERD **C** habitat of *C.cyanites* at Camacan, Bahia **D** habitat of two Brazilian males of *C.harrisi* sp. nov. (Photograph credit: Bruno Ferreira).

Figs 1 , 2A, B , 3A–D , 4A–D , 5 , 6A–C

Euptychia cyanites : Butler, 1871: 282–283 [original description]; Kirby 1877: 700; Weymer 1911: 218, pl. 49c; Gaede 1931: 444 [in the synonymy of Papiliocrantor Fabricius, 1793]; D’Abrera 1988: 766, figs; Lamas 2004: 217.
Caeruleuptychia sp.: Nakahara et al. 2018b: fig. 1C.
Caeruleuptychia sp. nov. 2: Nakahara et al. 2022: fig. 1.
Caeruleuptychia cyanites : Espeland et al. 2023: fig. 10.

#### Type locality.

Brazil. ***Lectotype* (designated herein)** (Fig. 2A, B): male with following labels written verbatim separated by double forward slashes: //Presented by J. J. Joicey Esq. Brit. Mus.1931-291. // E.cyanites Butl. type// Type// Braz// BMNH (E) 1267021// (NHMUK).

### ﻿= Euptychia stigmatica

Taxon classificationAnimaliaLepidopteraNymphalidae

Godman, 1905

80AE3DDE-4DA5-584F-B0B5-80EC338A002F

Fig. 2C–F

Euptychia stigmatica : Godman 1905: 186, pl. X[10], fig. 2 [original description]; Weymer 1911: 218; [in the synonymy of cyanites Butler, 1871]; Riley and Gabriel 1924: 55; Gaede 1931: 444 [in the synonymy of Papiliocrantor Fabricius, 1793]; D’Abrera 1988: 766 [in the synonym of E.cyanites]; Lamas 2004: 217 [in the synonymy of E.cyanites Butler, 1871].

#### Type locality.

“Entre Rios, Argentina” = Três Rios, Rio de Janeiro, Brazil (see discussion below). ***Holotype*** (fixed by monotypy) (Fig. 2C–F): male with following labels written verbatim separated by double forward slashes: //BMNH(E) 1267020//Type//Type of Species// Sept.//Entre Rios, Argentina H. H. Smith//Godman-Salvin Coll. 1904. – 1. Euptychiastigmatica, Godm.//B. M. TYPE No. Rh.3167 Euptychiastigmatica, ♂ Godm.//(NHMUK).

#### Systematic placement and diagnosis.

The genus *Caeruleuptychia* is found as a clade in the so-called *Splendeuptychia* clade of Euptychiina (Espeland et al. 2019; 2023). Within *Caeruleuptychia*, *C.cyanites* is recovered as a member of the *caerulea* clade based on the ML tree inferred from COI barcode data (Nakahara et al. 2022: fig. 1, as *Caeruleuptychia* sp. nov. 2). Despite the *caerulea* clade not being found as monophyletic in the present work, preceding studies (e.g., Nakahara et al. 2022) did recover this clade as a natural group. *Caeruleuptychiacyanites* is recovered as a sister taxon to *C.harrisi* sp. nov. with a strong support in the present work (Fig. 1A), where this clade (*C.cyanites* + *C.harrisi* sp. nov.) is found as a sister to *C.coelestis* (Butler, 1867), and furthermore, ((*C.cyanites* + *C.harrisi* sp. nov.) + *C.coelestis*) is recovered as a sister to (*C.aemulatio* sp. nov. + *C.glauca*) . The mean inter-specific pairwise distance between *C.cyanites* and *C.harrisi* sp. nov. was 1.6%, and mean infra-specific pairwise distance of 0.1% (*n* = 6) (*C.cyanites*) and 0.2% (*n* = 2) (*C.harrisi* sp. nov.).

Female specimens are currently known only for *C.cyanites* among taxa discussed herein, thus the diagnostic characters provided below are restricted to male individuals. *Caeruleuptychiacyanites* is distinguished from *C.harrisi* sp. nov. by its larger forewing length: 22.8–26.1 mm, mean 24.66 mm (*n* = 16) for *C.cyanites*; 21.0–22.4 mm, mean 21.38 mm (*n* = 5) for *C.harrisi* sp. nov. *Caeruleuptychiacyanites* is further distinguished from *C.harrisi* sp. nov. by possessing a larger scent patch in the dorsal forewing cell 2A, where this patch encompasses ca. one-third of the forewing inner margin (25–31%, mean 28.0% (*n* = 16)), whereas this patch encompasses less than one-fifth of the forewing inner margin (14–19%, mean 16.2% (*n* = 5)) in *C.harrisi* sp. nov. (see Table 1). The length of this dorsal forewing scent patch occupies ca. one-fourth of the forewing inner margin in *C.aemulatio* sp. nov. (21–27%, mean 23.7.4%, *n* = 6; Table 1). We created bivariate scatterplots based on these forewing measurements to graphically delineate these three species discussed above (Fig. 1B). *Caeruleuptychiacyanites* is further distinguished from *C.aemulatio* sp. nov. by often having the dorsal hindwing cell Cu_1_ adorned with a black spot, whereas this spot is absent in *C.aemulatio* sp. nov.; by having broader dark bands on the ventral surface (most pronounced in the ventral forewing umbra); and by cell Cu_2_ on the ventral forewing having paler blue scaling than the remainder of the wing, with this color extending slightly into the posterior half of cell Cu_1_, with the middle of cell Cu_2_ having even paler, whitish blue scales that might represent androconial scales, whereas similar pale whitish blue scales in *C.aemulatio* sp. nov. are confined to cell 2A. Nevertheless, given the subtlety of these characters and slight variation, it is advisable to use these characters in combination in order to distinguish *C.cyanites* from *C.aemulatio* sp. nov. *Caeruleuptychiacyanites* is further distinguished from *C.harrisi* sp. nov. by the ventral margin of the tegumen exhibiting an upwards concavity in lateral view, as well as an apical process of the valva terminating in a broader point in lateral view. In addition, the male of *C.coelestis* is readily distinguishable from these three taxa discussed above by the absence of a forewing scent patch.

#### Variation.

Black spot in the dorsal hindwing cell Cu_1_ is variable in male specimens in size, namely clearly present in many individuals although reduced or apparently absent in some specimens (e.g., a pair at FLMNH; lectotype of *E.cyanites*).

#### Examined material

**(21 males and 18 females). Males**: With the following labels written verbatim separated by double forward slashes: São Paulo – //Casa Br[anca]. G. (MfN); //Prov. S. Paulo Brasil mer. Littke.// Most probably ♂ of Euptychia ziza, Butl.// genitalia vial M-9125 ♂ Lee D. Miller (MfN); Minas Gerais – //P[arque]e[stadual do]r[io]d[oce] – 05/VIII/15 Trilha 2 – Arm 3D Lagoa Bonita// BLU 778 [molecular voucher]// (ZUEC); //BRAZIL, Minas Gerais, Timóteo Pq. Est. do Rio Doce 05.VIII.2015 Lourenço G.M., Soares G.R. & Palacio T. leg.// YPH-0854 [molecular voucher]// ZUEC-LEP 12054// (ZUEC); //BRAZIL, Minas Gerais, Timóteo Pq. Est. do Rio Doce 06.X.2015 Lourenço G.M., Soares G.R. & Palacio T. leg.// ZUEC-LEP 12055// (ZUEC); //BRAZIL, Minas Gerais, Timóteo Pq. Est. do Rio Doce 04.X.2015 Lourenço G.M., Soares G.R. & Palacio T. leg.// ZUEC-LEP 12064// (ZUEC); //BRAZIL, Minas Gerais, Timóteo Pq. Est. do Rio Doce 04.X.2015 Lourenço G.M., Soares G.R. & Palacio T. leg.// ZUEC-LEP 12056// (ZUEC); //BRAZIL, Minas Gerais, Timóteo Pq. Est. do Rio Doce 14.IX.2015 Lourenço G.M., Soares G.R. & Palacio T. leg.// ZUEC-LEP 12057// (ZUEC); //BRAZIL, Minas Gerais, Timóteo Pq. Est. do Rio Doce 12.IX.2015 Lourenço G.M., Soares G.R. & Palacio T. leg.// ZUEC-LEP 12058// (ZUEC); //BRAZIL, Minas Gerais, Timóteo Pq. Est. do Rio Doce 15.IX.2015 Lourenço G.M., Soares G.R. & Palacio T. leg.// ZUEC-LEP 12059// (ZUEC); //29-I–3-II-2003 . ESTAÇÃO BIOLÓGICA DE CARATINGA, CARATINGA, MG, 400 m MIELKE & CASAGRANDE LEG.// (DZUP); Espírito Santo – //BRAZIL, Espírito Santo, Sooretama, Reserva Biológica de Sooretama, 19°02'07.0"S, 40°09'30.2"W, 18.VIII.2018, Santos J.P., Rosa A.H.B., Machado P.A. & Lopes A.C. leg.// LBR0654 [molecular voucher]// (ZUEC); //ESPÍRITO SANTO RES. ECOLÓGICA SOORETAMA 19°03'25"S, 40°08'50"W 19-26 – II – 2013 MIELKE & CASAGRANDE LEG.// DZ 40.523// BC-DZ Willmott 8 [molecular voucher]// (DZUP); BRAZIL, ESPÍRITO SANTO RES. ECOLÓGICA SOORETAMA 19°03'25"S, 40°08'50"W 19-26 – II – 2013 MIELKE & CASAGRANDE LEG.// DZ 40.372// BC-DZ Willmott 6 [molecular voucher]// (DZUP); BRAZIL, ESPÍRITO SANTO RES. ECOLÓGICA SOORETAMA 19°03'25"S, 40°08'50"W 19-26 – II – 2013 MIELKE & CASAGRANDE LEG.// DZ 40.373// (DZUP); BRAZIL: ESPIRITO SANTO: Itaguassu 1x.1971 Paulo Cesar Elias// A. C. Allyn Acc. 1971-38// Genitalic vial SN-19-13// Allyn Museum photo No. 073075-11// FLMNH 1138898// (FLMNH); BRAZIL, BAHIA, CAMACAN RESERVA SERRA BONITA 15°23'S, 39°33'W, 40°08'50" 3–10-XII-2016 200 m MIELKE, CARNEIRO, DIAS, DOLIBAINA & SANTOS LEG.// DZ 40.512// (DZUP); BRAZIL, BAHIA, CAMACAN RESERVA SERRA BONITA 15°23'S, 39°33'W, 40°08'50" 3–10-XII-2016 200m MIELKE, CARNEIRO, DIAS, DOLIBAINA & SANTOS LEG.// DZ 40.503// (DZUP); BRAZIL, BAHIA, CAMACAN RESERVA SERRA BONITA 15°23'S, 39°33'W, 40°08'50" 3–10-XII-2016 200m MIELKE, CARNEIRO, DIAS, DOLIBAINA & SANTOS LEG.// DZ 40.502// BC-DZ Willmott 9 [molecular voucher]// (DZUP); //CONCEIÇÃO BARRA ES – BRASIL 10-IX-1969 C.& C.T.Elias lg// GEN. PREP. BILOTA 1997// DZ 5268 ♂ Caeruleuptychiacyanites I. G. BILOTA DET. 1997// DZ 5.268// (DZUP); Bahia – //Rio. R. Hewitson Coll. 79–69. Euptychia Coelestis Butl. 1.// BMNH(E) 1717835// (NHMUK). **Females**: with the following labels written verbatim separated by double forward slashes: Rio de Janeiro – //ALCINDO GUANA-BARA-E.F.C.B. 15-IV-1940 P. SANDIG LEG// Ex Col. Gagarin// alcindo guanabara E.F.C.B. Paulo Sandig 15-4-1940// DZ 40.381// (DZUP); //Novo Friburgo.// genitalia vial M-9126 ♀ Lee D. Miller// coll. Sommer (MfN); Minas Gerais – BRAZIL, Minas Gerais, Timóteo Pq. Est. do Rio Doce 05.X.2015 Lourenço G.M., Soares G.R. & Palacio T. leg.// ZUEC-LEP 12063// (ZUEC); //BRAZIL, Minas Gerais, Timóteo Pq. Est. do Rio Doce 15.IX.2015 Lourenço G.M., Soares G.R. & Palacio T. leg.// ZUEC-LEP 12062// (ZUEC); //BRAZIL, Minas Gerais, Timóteo Pq. Est. do Rio Doce 06.XI.2015 Lourenço G.M., Soares G.R. & Palacio T. leg.// ZUEC-LEP 12061// (ZUEC); //BRAZIL, Minas Gerais, Timóteo Pq. Est. do Rio Doce 11.V.2016 Lourenço G.M., Soares G.R. & Palacio T. leg.// ZUEC-LEP 12060// (ZUEC); Espírito Santo – //BRAZIL, ESPÍRITO SANTO RES. ECOLÓGICA SOORETAMA 19°03'25"S, 40°08'50"W 19–26 – II – 2013 MIELKE & CASAGRANDE LEG.// (DZUP); BRAZIL: E. SANTO Baixo Guandu x.1971 P. C. Elias// A. C. Allyn Acc. 1971-47// Genitalic vial SN-19-14// Allyn Museum photo No. 073075-10// (FLMNH); //BRAZIL, ESPÍRITO SANTO RES. ECOLÓGICA SOORETAMA 19°03'25"S, 40°08'50"W 19–26 – II – 2013 MIELKE & CASAGRANDE LEG.// DZ 40.522// (DZUP); //BRAZIL, ESPÍRITO SANTO RES. ECOLÓGICA SOORETAMA 19°03'25"S, 40°08'50"W 19–26 – II – 2013 MIELKE & CASAGRANDE LEG.// DZ 48.799// BC-DZ Willmott 7 [molecular voucher]// (DZUP); //BRAZIL, ESPÍRITO SANTO RES. ECOLÓGICA SOORETAMA 19°03'25"S, 40°08'50"W 19–26 – II – 2013 MIELKE & CASAGRANDE LEG.// DZ 40.513// (DZUP); //BRAZIL, ESPÍRITO SANTO RES. ECOLÓGICA SOORETAMA 19°03'25"S, 40°08'50"W 19–26 – II – 2013 MIELKE & CASAGRANDE LEG.// DZ 48.819// (DZUP); //BRAZIL, ESPÍRITO SANTO RES. ECOLÓGICA SOORETAMA 19°03'25"S, 40°08'50"W 19–26 – II – 2013 MIELKE & CASAGRANDE LEG.// DZ 48.809// (DZUP); //BRAZIL, ESPÍRITO SANTO RES. ECOLÓGICA SOORETAMA 19°03'25"S, 40°08'50"W 21–25 – I – 2014 MIELKE & CASAGRANDE LEG.// DZ 40.363// (DZUP); //BRAZIL, ESPÍRITO SANTO RES. ECOLÓGICA SOORETAMA 19°03'25"S, 40°08'50"W 19–26 – II – 2013 MIELKE & CASAGRANDE LEG.// DZ 40.351// (DZUP); //Conceição da Barra Esp[írito]. Santo Brazil 18-VI-1969 C. & C. T. Elias leg.// DZ 40.352// (DZUP); //CONCEIÇÃO BARRA ES – BRASIL 18-X-1968 C.& C.T.Elias leg// DZ 5.269// DZ 5269 ♀ Caeruleuptychiacyanites I. G. BILOTTA DET. 1997// GEN. PREP. BILOTTA 1997// (DZUP); Bahia – //BRASIL: BA, Camacan Res. Serra Bonita 15°23'S–39°33'W 800m, xi. 2012 V.O. Becker Col.// (VOB); Rio. Hewtson Coll. 79–69. Euptychia Coelestis Butl. 2. [“Rio” on the underside]// BMNH(E) 1717836// (NHMUK).

#### Other examined material

**(3 females).** With the following labels written verbatim separated by double forward slashes: São Paulo (?) – Araras (S.P.) 600 m, 11.11.65 Ebert// Colecão H.Ebert// DZ 40.371//(DZUP); //Araras (S. Paulo) 600 m 23.11.68 Ebert// Colecão H.Ebert// DZ 40.361//(DZUP); //Araras (S. Paulo) 600 m 23.11.68 Ebert// Colecão H.Ebert// DZ 40.362//(DZUP).

#### Distribution

**(Fig. 5).** This species is known from the states of Bahia, Espírito Santo, Minas Gerais, Rio de Janeiro, and São Paulo, all situated in southeastern Brazil. Records from Araras, São Paulo are likely erroneous.

#### Biology

**(Fig. 6).** Based on recent records, *C.cyanites* is associated with areas of well-preserved old growth Atlantic Forest. In a 12-month trap study in PERD, Minas Gerais, a total of 34 individuals were captured, 26 captured in the canopy and 8 in the understory, indicating a clear preference for the upper stratum (X^2^ = 9.529, P < 0.0020, DF = 1). The sex could be attributed to 33 of the captured individuals, totaling 18 males and 15 females, a sex ratio not significantly different from 1:1 (1:0.83, X^2^ = 0.27, P = 0.6015, DF = 1). Most records (32 of 34) were concentrated from August to November/2015. In addition, most individuals (*n* = 20) were captured in the ecotone between forests and lakes, a habitat with high sunlight availability resulting in the formation of a “brought low canopy” (5–15 m high); the remaining individuals were captured in the forest interior (*n* = 13) and a single individual was captured in the forest edge. General behavior, immature stages and host plants are unknown.

### ﻿ Caeruleuptychia harrisi

Taxon classificationAnimaliaLepidopteraNymphalidae

Nakahara & Freitas sp. nov.

DA07E33D-EE7F-54ED-82FB-165992DE3481

https://zoobank.org/D941C073-4AA4-4268-AB81-570F064ABA47

Figs 1 , 3E, F , 4E, F , 5 , 6D

Caeruleuptychia cyanites 01: Nakahara et al. 2018b: fig. 1C.; Espeland et al. 2023: fig. 10.
Caeruleuptychia sp. nov. 1: Nakahara et al. 2022: fig. 1.

#### Systematic placement and diagnosis.

See corresponding section of *C.cyanites* for information regarding systematic placement and diagnostic characters for this taxon.

#### Description.

**Male: *Head***: Eyes with short hair-like setae, with white scales at base; frons and vertex black; first segment of labial palpi adorned with long white hair-like scales, second segment length approximately twice eye depth and covered with white hair-like scales and white scales laterally, and with brownish hair-like scales along edge of distal two-thirds of dorsal surface, ventrally adorned with black hair-like scales and white hair-like scales longer than segment width, third segment porrect, approximately one-third of second segment in length and covered with black scales dorsally and ventrally, with whitish scales laterally; antennae approximately two-fifths of forewing length, with ca. 37 segments (*n* = 1), distal 12 or 13 segments composing insignificant club. ***Thorax***: Dorsally and laterally scattered with whitish scales, brownish scales and bluish scales (whitish scales visible towards base of wing, brownish scales visible anteriorly and bluish scales visible posteriorly), lightly colored long hair-like scales visible posteriorly, ventrally scattered with brownish scales with bluish scales visible on metathorax; prothoracic leg with whitish scales and brownish scales, in addition to whitish long hair-like scales and brownish hair-like scales, femur, tibia and tarsus almost same in length; pterothoracic legs femur covered with short creamy scales, dorsally darker; tarsus and tibia grayish, dorsally darker, adorned with few longitudinal rows of spines ventrally, row of lateral spines present on inner side of tibia, tibial spurs present at distal end of tibia. ***Abdomen***: Eighth tergite appearing sclerotized along basal margin of dorsal surface of eighth abdominal segment. ***Wing venation***: Basal half of forewing subcostal vein swollen; base of cubitus swollen; forewing recurrent vein absent; hindwing humeral vein developed; origin of M_2_ slightly towards M_1_ than M_3_. ***Wing length and shape***: 20.8–22.6 mm, mean 21.38 mm (*n* = 5); forewing subtriangular, appearing somewhat elongate and pointy, apex rounded, costal margin convex, outer margin almost straight, inner margin straight, but rounded towards thorax near base; hindwing slightly elongate, rounded, costal margin convex, outer margin slightly sinuate, inner margin slightly concave near tornus, anal lobe convex, slightly rounded. ***Dorsal forewing***: Distal ca. one-fifth with brownish scales, apparently narrowing in width from apex towards tornus, remaining area powder blue, brownish scales along m_1_-m_2_, discal band appearing as inconspicuous short brownish scaling in discal cell, concolorous postdiscal band broader and more defined, extending from radius to cell Cu_2_ and tapering and terminating in this cell; elongate whitish/grayish scent patch positioned towards distal side in cell 2A compared to mid-point of cell 2A (occupying less than one-fifth of inner margin length), mosaic of pale and dark scales rounded at tips (compared to more flat-tipped scales of adjacent region), feather-like androconial scales absent; fringe grayish. ***Dorsal hindwing***: Area mainly anterior of M_1_ and distal side of cell M_1_ brownish, remaining wing surface basically powder blue; submarginal line, somewhat indistinct, extending from apex towards tornus, but fading before reaching cell 2A; concolorous marginal line, more defined, sinuate, traversing along outer margin; fringe grayish. ***Ventral forewing***: Ground color powder blue; brownish discal band extending from near costa, terminating at origin of Cu_2_; concolorous postdiscal band, wider than discal band, extending from near costa towards Cu_2_, and terminating around this vein; one submarginal ocellus in cell M_1_, appearing as black spot ringed with sky blue, pupil not confidently identified; umbra present as irregular, concolorous band extending from near costa to cell Cu_1_; concolorous submarginal band, extending from apex towards tornus, broadening in middle (between M_2_ and Cu_2_), overall smooth, terminating around 2A; concolorous marginal line, narrower than submarginal band, almost parallel with submarginal band and outer margin, somewhat broadening in cell Cu_2_; pale powder blue modified scales visible in middle of cell Cu_2_; fringe grayish. ***Ventral hindwing***: Similar to ventral forewing except as follows: bands appearing broader, short straight band present at base of ventral hindwing; discal band extending down to inner margin and passing basal of origin of Cu_2_; postdiscal line passing origin of Cu_1_ and extending down to inner margin; submarginal band bent in cell Rs and along M_1_, posterior end apparently occasionally fused with postdiscal band in cell 2A; marginal band jagged and extends along inner margin; five submarginal ocelli (but also see below), those in cells Rs, M_1_ and Cu_1_ appearing as a black spot ringed with sky blue, pupil may or may not be visible (see below), remaining ocelli appearing smaller, sky bluish smudge without black central area. ***Genitalia*** (Fig. 3E, F): Tegumen semi-circular in lateral view, anteriorly convex, dorsally convex but not as convex as anterior margin, ventral margin rather straight; uncus twice as long as tegumen in lateral view, hair-like setae visible dorsally and ventrally, base of uncus broadening in both lateral and dorsal view, tapering posteriorly with a concavity along dorsal margin in lateral view, and terminating in a hooked single point; brachium tapering towards apex, apical point positioned above uncus in lateral view, parallel to uncus with apical edge curving inwards in dorsal view; combination of ventral arms from tegumen and dorsal arms from saccus sinuate, broadening near saccus with up and down concavity; appendices angulares present, curving inwards; saccus straight, anteriorly somewhat rounded, shorter than uncus in length but longer than tegumen; juxta present as shallow U-shaped plate in posterior view; valva distal half setose, valva appearing roughly as parallelogram in lateral view, ventral margin convex, dorsal margin distal of costa accompanying hump at base of apical process, apical process terminating somewhat in thumb-like shape; phallus nearly straight, similar in length to tegumen plus uncus, phallobase less than half of phallus, ductus ejaculatorius visible as illustrated, posterior portion of aedeagus slightly curved upwards, manica not examined, cornuti visible as weakly sclerotized region of vesica. ***COI barcode***: Molecular vouchers: MGCL-LOAN-561 (GenBank voucher: MF192681); MGCL-LOAN-546 (GenBank voucher: MF192717).

**Female**: Unknown or unrecognized.

#### Variation.

The ventral hindwing ocellus in cell Rs is present in three specimens (FLMNH-MGCL-279345, MGCL-LOAN-546 and DZ 57.996), whereas it is absent in one specimen (MGCL-LOAN-561).

#### Etymology.

This specific epithet is in recognition of Brian P. Harris, for his tireless effort in facilitating butterfly research at USNM by going above and beyond to support visiting researchers. The holotype of *C.harrisi* sp. nov. was collected by Brian and deposited at USNM where he served as a museum specialist since 2005. The species-group name *harrisi* is considered to be a Latinized masculine noun in the genitive case.

#### Types.

***Holotype***: male with the following labels written verbatim separated by double forward slashes: //BRASIL: Rondonia 62 km S Ariquemes Faz.Rancho Grande 165 m 10.53°S, 62.80°W. 19–29. Sept.1996. B.Harris// Euptychiacyanites ♂// DNA voucher LEP-18635// USNM ENT 01844280// (USNM).

***Paratypes***: four males, with the following labels written verbatim separated by double forward slashes: //BRAZIL: RONDONIA Jaru ♂ .viii.1976 C. Callaghan// DNA voucher LEP-68762// FLMNH-MGCL Specimen 279345// Genitalic vial SN-17-35 S. Nakahara// A. C. Allyn Acc. 1976-15// (FLMNH) //Caiçara, Porto Velho, RO, BR 11.IX.2012 C1P4 150 m PMSF AHE Jirau MGCL 546// MGCL-LOAN-546// ZUEC-LEP 12053 [collected by Marcio Uehara-Prado]// (ZUEC); //Caiçara, Porto Velho, RO, BR 18.VI.2012 C1P4 250 m PMSF AHE Jirau MGCL 561 [collected by Marcio Uehara-Prado]// MGCL-LOAN-561 [molecular voucher]// MGCL-561 [genitalia dissection label]// ZUEC-LEP 12052// (ZUEC); //N. 19.314// 7.II.1961. Vila Rondonia Rio Gi-parana. Terr. Rondonia. Angelo M. & P. Pereira [deposited in]III.1961 ♂// Coll. D’Almeida// DZ 57.996// (DZUP).

#### Distribution

**(Fig. 5).***Caeruleuptychiaharrisi* sp. nov. is known to date from the state of Rondônia, Brazil.

#### Biology.

Unknown, but see Fig. 6D for habitat image.

### ﻿ Caeruleuptychia aemulatio

Taxon classificationAnimaliaLepidopteraNymphalidae

Nakahara & Willmott sp. nov.

6D8EBF9F-0163-5B16-BDDE-E174708922DB

https://zoobank.org/0BE4FDF8-DD0A-4A8B-879B-FFD1243D1706

Figs 1 , 3G, H , 4G, H , 5

Caeruleuptychia cyanites : Nakahara et al. 2018b: fig. 1.; Nakahara et al. 2022: fig. 1.; Espeland et al. 2023: fig. 10.

#### Systematic placement and diagnosis.

*Caeruleuptychiaaemulatio* sp. nov. is recovered as sister to *C.glauca* with a moderate support based on the COI data presented herein (Fig. 1A). This clade (*C.aemulatio* sp. nov. + *C.glauca*) is found as sister to (*C.coelestis* + (*C.harrisi* sp. nov. + *C.cyanites*) with a strong support (Fig. 1A). *Caeruleuptychiaaemulatio* sp. nov. is readily distinguishable from *C.harrisi* sp. nov. by its larger dorsal forewing scent patch (see corresponding section of *C.cyanites*, as well as Table 1 for further details), in addition to lacking pale modified scales on the ventral forewing cell Cu_2_. The ventral bands overall are narrower in *C.aemulatio* sp. nov., and the rings of the ventral hindwing ocelli in cells M_1_ and Cu_1_ are broader in *C.aemulatio* sp. nov. compared to the immediately preceding species.

#### Description.

Largely in accordance with the immediately preceding species with differences documented above. ***COI barcode***: Molecular vouchers: MGCL-LOAN-452 (GenBank voucher: MF192713); MGCL-LOAN-447 (GenBank voucher: MF192712).

#### Variation.

The smudge-like ocelli lacking a black central area in the ventral hindwing of cells M_2_ and M_3_ are clearly visible in the holotype specimen from Cuzco department, Peru (housed at USNM), whereas they are rather insignificant in the specimen from Rio “Pachytera” [= Pachitea], [Huánuco], Peru (housed at FLMNH).

#### Etymology.

This new species-group name is a feminine Latin noun meaning “emulation” or “desire to equal or excel others”, in reference to the resemblance and relatedness of this new species to two other *Caeruleuptychia* species discussed herein.

#### Types.

***Holotype***: male with the following labels written verbatim separated by double forward slashes: PERU, MD, Reserva Comunal Amarakaeri, Río Azul 507 m 1249/7106 11.x.2010 M. Vílchez// MUSM-LEP 100094// MUSM Loan KW-15-086// Photographed By K. Willmott June 2015// (MUSM). ***Paratypes***: eleven males, with the following labels written verbatim separated by double forward slashes: //Foz do Rio Tejo, Estrada para o Rio Arara, Res. E. A. J., AC BR 16.IX.1997 Brown Junior, K. S., Freitas, A. V. L. MGCL 447// MGCL-LOAN-447 [molecular voucher]// (ZUEC); //Foz do RioBreu, Res. Extravitista do Alto Juruá, AC, BR 12.IX.1999 Brown Junior, K. S., Freitas, A. V. L. MGCL 452// MGCL-LOAN-452 [molecular voucher]// (ZUEC); //PERU: Cuzco, 540 m Pilcopata, Villa Carmen Cosñipata Valley 6923 31-1-2020 Kinyon// Genitalic vial SN-MCZ-003//(USNM); //Est Pérou [East Peru] Rio Pachytera [Pachitea] Coll. Le Moult// Slide No. M-2985 ♂ genitalia Lee D. Miller// FLMNH MGCL 1138899//(FLMNH); //Tambopata – Candamo Reserve, Peru 1/81 ♂// Genitalic vial SN-17-34 S. Nakahara// J. Brenner coll. MGCL Accession #2015-33// FLMNH-MGCL Specimen 279343// (FLMNH); 28–30- I – 2009 RESERVA HUMAITA, PORTO ACRE ACRE, MIELKE & CASAGRANDE LEG 09°73'S, 67°68'W// DZ 57.995// (DZUP); //8-10-IX 2004 RESERVA HUMAITA, PORTO ACRE ACRE, 200 m, O. MIELKE & CASAGRANDE LEG// BC-DZ Willmott 19 [molecular voucher]// DZ 57.994// (DZUP); //PERU, MD, Boca Rio La Torre 300 m 19.x.85 G. Lamas// MUSM-LEP 100090// (MUSM); PERU: Madre de Dios Manu, Pakitza, 340 m 11°55'48"S, 71°15'18"W 12 May 1991 leg. D. J. Harvey// MUSM-LEP 100092// Genitalic vial SN-17-86 S. Nakahara// (MUSM); // PERU: Madre de Dios Parque Manu, Pakitza 11°53'S, 70°58'W, 400 m 6 Oct 1990 leg. R. Robbins// MUSM-LEP 100093// PERU, MD, Albergue Amazonia 1252/7123 500 m 28.x.2010 G. Lamas// MUSM-LEP 100095// MUSM Loan KW-15-089// (MUSM).

#### Distribution.

*Caeruleuptychiaaemulatio* sp. nov. is known to date from the state of Acre, Brazil, as well as Huánuco, Cuzco, and Madre de Dios departments in Peru.

#### Biology.

Unknown.

## ﻿Discussion

Application of species-group names (specific vs subspecific) to a sister pair of allopatric taxa involves some degree of subjectivity. Therefore, the species-level status of *C.cyanites*, *C.harrisi* sp. nov., and *C.aemulatio* sp. nov. can be challenged considering their low genetic divergence and allopatric distribution, coupled with their rather trivial differences in adult external morphology. Nevertheless, we consider our conclusions to be justifiable based on the following arguments provided herein. One principal morphological character to consider these three taxa to be accorded specific status is the size difference in the dorsal forewing androconial scent patches. The taxonomic value of this androconial scent patch can be inferred from two closely related species in the *caerulea* clade, *C.coelestis* and *C.aemulatio* sp. nov. Male specimens of *C.coelestis* lack the androconial scent patch, whereas this feature is present in *C.aemulatio* sp. nov. These two species are broadly sympatric in the southwestern Amazonia and their species-level status is delineated in the phylogenetic structure (Fig. 1A), with mean inter-specific pairwise distance of 4.0% based on the dataset used in the present study. Additionally, Nakahara et al. (2022) described *C.thaliana* Nakahara & Piovesan, 2022 on the grounds of broad sympatry with its sister species, *C.umbrosa* Butler, 1870, in conjunction with the presence/absence of the dorsal forewing androconial hair-pencil, with support from molecular data. Although the role of these androconial scales are not fully explored in euptychiines, a wealth of research has been conducted on another Satyrinae genus, *Bicyclus* Kirby, 1871 (Satyrini: Mycalesina): Condamin (1973) implied that the androconial scales on wings are informative species-level diagnostic characters in *Bicyclus* by providing such information in his dichotomous key, and subsequent studies revealed that the male sex pheromone component released by sympatric species pairs displays larger differences compared to compounds emitted by an allopatric pair of *Bicyclus* species (e.g., Bacquet et al. 2015). These studies on *Bicyclus*, coupled with the differences observed in sympatric *Caeruleuptychia* sister species pairs, suggest that these androconial scales can potentially play a key role in interspecific reproductive isolation in euptychiines as well. This conclusion reinforces our use of the size difference observed in the dorsal forewing androconial scent patch between *C.cyanites*, *C.harrisi* sp. nov. and *C.aemulatio* sp. nov. as a species-level diagnostic character. Furthermore, these three species are found separately in Amazonia (*C.harrisi* sp. nov. and *C.aemulatio* sp. nov.) and the Atlantic Forest (*C.cyanites*), two of the four major biogeographic regions in the Neotropics. As mentioned above, these two biogeographical regions are separated by a diagonal of open vegetation formations, currently with no known contact zone, and thus potentially leading to different biological traits. Indeed, *C.cyanites* appears to be a canopy dwelling species, which is an unusual feature for an euptychiine species, since members of this subtribe are often encountered in the understory of the forest (pers. obs., but see Singer et al. 1983 and Freitas et al. 2021 for discussions of other euptychiine species with similar canopy habits). With no equivalent trait data for *C.harrisi* sp. nov. and *C.aemulatio* sp. nov., it would be premature to develop the discussion further, but we consider the species-level classification introduced herein to be informative based on the aforementioned evidence, as well as taking into account the genetic data with a clear barcoding gap (Fig. 1A). The uniting of *C.cyanites* and *C.harrisi* sp. nov. and regarding one as a subspecies of the other might be more parsimonious, although the existence of relatively few euptychiine species known to occur in both Amazonia and the Atlantic Forest (e.g., *Amigaarnaca* (Fabricius, [1777])), argues against such an approach.

*Euptychiacyanites* was described by Butler (1871: 282–283) based on an unspecified number of male specimen(s) from Brazil in [William Wilson] Saunders’ collection. The sex is not explicitly stated in the original description, although the mention of “the curious scaly whitish patch on the interior margin of the anterior wings on the upperside” in the diagnosis clearly points to the examined specimen(s) being a male. The male syntype of *E.cyanites* (Fig. 2A, B) is housed at NHMUK and the label information is provided above. The forewing length of this syntype is 25 mm (the right forewing is angled away and appears distorted in Fig. 2A, B), which falls within the forewing length range of *C.cyanites* (see Table 1). Other notable features include a large scent patch on the dorsal forewing in cell 2A, which is larger than that of *C.harrisi* sp. nov. and *C.aemulatio* sp. nov. (see Table 1), as well as the presence of pale powder-blue modified scales visible in the middle of the ventral forewing cell Cu_2_. These characteristics suggest that the species name *cyanites* cannot be applied to concepts represented by the two other species-group names introduced in the present work, but applies to individuals of a *Caeruleuptychia* species known from the Atlantic Forest. Although the type locality of *E.cyanites* was only stated as “Brazil” with no further information as to its provenance, evidence suggests William Saunders’ collection did hold materials from southeastern Brazil (Hewitson 1858). Additionally, for an unknown reason, Gaede (1931: 444) associated Minas Geraes [Gerais] as the locality for *cyanites*, thus we have some support besides external morphology to narrow down the identity of *E.cyanites*.

*Euptychiastigmatica* Godman, 1905 was described by Frederick DuCane Godman based on a single male specimen from “Entre Rios, Argentina” collected by H[erbert] H[untingdon] Smith (Godman 1905: 186). The statement of one specimen in the original description enables us to regard this male specimen as a holotype fixed by monotypy following Article 73.1.2 of the ICZN (1999). In addition to this holotype, Godman (1905) mentions the existence of specimens in the British Museum [=NHMUK] of what he considered as a conspecific pair from “Rio River”. The transcribed label data for these two specimens housed at the NHMUK are provided above. Butler (1869: 347) stated that “Rio R.” of [William Chapman] Hewitson probably refers to “Rio Real” in Bahia, Brazil. This interpretation of “Rio R.” representing a site situated in Bahia, Brazil falls within the range (i.e., Atlantic Forest) of *C.cyanites* and the whereabouts of Godman’s “Rio River” is unknown. Subsequently, Weymer (1911: 218) regarded *E.stigmatica* to be a junior subjective synonym of *E.cyanites*. Gaede (1931: 444) considered both *E.cyanites* and *E.stigmatica* to be synonymous with *Papiliocrantor*, although with no justification. Gustav Weymer also did not provide rationale for his synonymy and referred to the type locality of *E.stigmatica* as “Entre Rios, Argentina”, in addition to stating that [Richard] Haensch collected both sexes [of *E.cyanites*] in Minas Gerais (Weymer 1911). The illustration accompanying the original description on pl. X, fig. 2 (Fig. 2D, E) clearly shows a large scent patch in the dorsal forewing cell 2A. This feature is also visible in the male holotype housed at the NHMUK (Fig. 2C, D; see above for transcribed label data), which readily excludes this specimen representing many other species in the *caerulea* clade of *Caeruleuptychia*, including *C.harrisi* sp. nov. and *C.aemulatio* sp. nov. (see above). The scent patch length/inner margin length of this male holotype is 27%, which is greater than that of *C.harrisi* sp. nov. (14–19%, mean 16.2%) *C.aemulatio* sp. nov. (21–27%, mean 23.7%) and in accordance with *C.cyanites* (25–31%, mean 28%). Despite the lack of dorsal hindwing black spot in cell Cu_1_ and pale powder-blue scales on the ventral forewing in cell Cu_2_ not being discernible (due to overlap with the hindwing?), the forewing length of this holotype is 25.4 mm, which supports its identity as *C.cyanites*, thus we continue the discussion by following the footsteps of the remarkable naturalist who collected the holotype of *E.stigmatica*.

**Figure 7.**
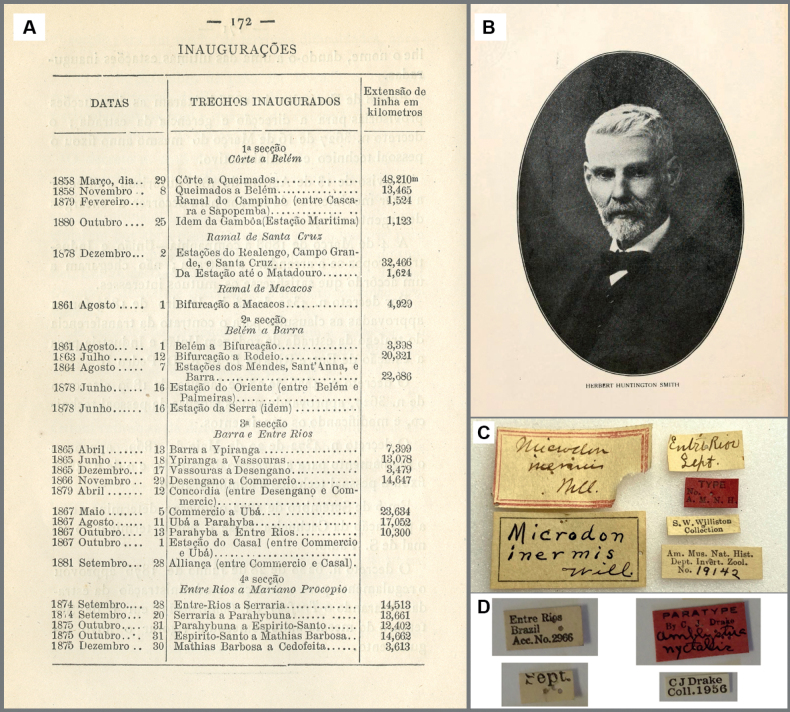
**A** table from Picanço (1884: 172) showing Estrada de Ferro D. Pedro II reaching Entre Rios (= Três Rios) in 1867 (image reproduced from Harvard Business School (HBS) Archives, Baker Library, HBS at Harvard University) **B** portrait of Hebert H. Smith from volume 30 of The Nautilus, where Clapp’s (1920) article appeared **C** labels associated with the holotype of *Microdoninermis* (Diptera) (AMNH) **D** labels associated with the paratype of *Amblystiranyctalis* (Hemiptera) (USNM).

Herbert Huntingdon (or Huntington) Smith (1851–1919) (Fig. 7B) was an American naturalist, as well as being known as a conchologist later on in his life, who traveled throughout tropical America collecting faunistic and floristic materials (Holland 1919; Clapp 1920). Smith was first introduced to the delights of the Neotropical fauna and flora in 1870, when he traveled to the Brazilian Amazon as a member of the Morgan expeditions with Charles Frederick Hartt (Smith 1879a; Kunzler et al. 2011). In 1874, Smith returned to Brazil and spent two years around Santarém (Pará, Brazil) (Smith 1879a), followed by a year exploring the northern tributaries of the Amazon basin and the Tapajós, and returned to the United States after residing for four months in Rio de Janeiro. He was then able to make additional trips to Brazil supported by the publishing firm Messrs Scribner & Co., and Smith wrote six articles for their Scribner’s Monthly journal, based on his revisit to Brazil in return (Kunzler et al. 2011). One of these articles was entitled “Coffee culture in Brazil” (Smith 1879b), which also appeared in chapter 18 (The Story of Coffee) in Smith’s (1879a) “Brazil the Amazon and the coast”, where he reported his studies on the coffee industry and referred to the locality he visited as “Entre Rios”. In his introduction of this site, Smith (1879a, b) stated that “the story begins on the hills around Entre Rios, away back of [from] the Organ mountains”. The mention of Organ mountains [= Serra dos Órgãos] implies that the place named “Entre Rios” in Smith (1879a, b) is located in the vicinity of Rio de Janeiro, Brazil, already contradicting the type locality of *E.stigmatica* stated by Godman (1905) as “Entre Rios, Argentina”. Smith (1879a: 530) further refers to his Entre Rios as “Entre Rios is on a branch of the Dom Pedro Segundo Railroad [Estrada de Ferro D. Pedro II], where the latter meets the Uniao e Industria [Estrada de Rodagem União e Indústria]”. Estrada de Ferro D. Pedro II departed from Côrte [= Rio de Janeiro?] on March 29, 1858 and reached Entre Rios, a municipality in Rio de Janeiro currently known as Três Rios, on October 13, 1867 (Fig. 7A; Picanço 1884: 172). The inauguration of Estrada de Rodagem União e Indústria took place in 1861 (Bartholomeu 2019), and connected Petrópolis (Rio de Janeiro) and Juiz de Fora (Minas Gerais). Bartholomeu (2019: fig. 2) shows passing of Estrada União e Indústria through Três Rios [= Entre Rios], which corresponds to Smith’s statement “Entre Rios is on a branch of the Dom Pedro Segundo Railroad, where the latter meets the Uniao e Industria”, since this is the only place represented by the name Entre Rios reached by both the União e Indústria and Estrada de Ferro D. Pedro II. Smith (1879a: 511) started his “The Story of Coffee” by stating “In 1878, Brazil exported more than five hundred million pounds of coffee.” Thus, assumption can be made that Smith was studying the coffee industry in Entre Rios at some point in 1878. In the chapter entitled “Ceará and the Drought” in his “Brazil…coast”, Smith (1879a: 421) wrote “I reached Fortaleza [Ceará, Brazil] on 19^th^ of that month [December 1878]”, and based on his (seemingly) chronologically written preface of “Brazil…coast”, he likely studied the coffee industry prior to his travel to Ceará. De Abreu (1922) also stated that Smith passed through Ceará and Pernambuco on his way back to the USA, therefore, he likely traveled to eastern Brazil in 1878, towards the end of his journey. If this holds true, Smith was in Entre Rios to study the coffee culture prior to December 1878. This assumption is in accordance with the printed label “Sept. [year not indicated]” associated with the holotype of *E.stigmatica*. It is worth noting that a species of a fly, *Microdoninermis* Williston, 1888 (Diptera: Syrphidae), was also described based on a single specimen collected by H. H. Smith in “Entre Rios” in September [year not indicated] (Williston 1888: 258). Despite the referral of its type locality to Argentina in subsequent catalogs (e.g., Thompson et al. 1976), the labels associated with the type specimen (i.e., holotype, housed at AMNH) of *M.inermis* bear no label indicating the country of origin, except for a handwritten label “Entre Rios Sept.”, in accordance with the original description (Fig. 7C). This observation raises the likelihood that the holotype of *M.inermis* was collected by Smith in September of an unknown year when he also sampled the holotype of *E.stigmatica* in Entre Rios, currently known as Três Rios (Rio de Janeiro, Brazil), perhaps in 1878.

Nevertheless, Smith explored the Brazilian Amazon again, by signing a contract with Ladislau de Souza Mello e Netto (then director of the MNRJ) at the end of 1881 (Doc. MN 237, folder 20, of 12/23/1881). In particular, Smith stayed in Mato Grosso for four years and collected extensively in “Chapada” [= Chapada dos Guimarães, Mato Grosso, Brazil] during this period (de Abreu 1922), until his return to the United States in 1886. His entomological samples of an estimated 10,000 insect species collected in Chapada yielded many species then unknown to science.

Smith’s (1922) “Do Rio de Janeiro a Cuyabá”, accompanied with a preface by Capistrano de Abreu, provides various records of Smith’s trips in Brazil and neighboring countries between 1881 and 1886. It is worth highlighting that Smith refers to a trip to Buenos Aires in chapter 19 of his “Do Rio de Janeiro a Cuyabá” (Smith 1922). Smith apparently traveled from Corumbá (in Mato Grosso) to Buenos Aires, via the Paraná River, consequently passing through Entre Rios province in Argentina. The details of his boat trip to Buenos Aires are not clear from the passage, but he did mention a visit to the Natural History Museum in Buenos Aires where he examined the extensive collection of Argentinean insects. Despite no mention of Entre Rios throughout over 350 pages in Smith (1922), it is therefore theoretically possible for him to have collected in Entre Rios province in Argentina on his way to Buenos Aires. This possibility is supported by the fact that Williston (1888: 249) examined dipteran specimens from “Corumbá” and “Rio Parana, near Bella Vista [Corrientes province, Argentina]” collected by Smith, likely during his trip to Buenos Aires via the Paraná River.

Smith returned to the United States in 1886 and his insect samples, including over 30,000 Lepidoptera specimens, were partly acquired by F. D. Godman in London (UK) and the Carnegie Museum of Natural History (USA) (Kunzler et al. 2011). Subsequently, numerous taxa were named and described based on these materials brought back by Smith: Godman (1905) described *Pierellachalybaea* Godman, 1905 (Nymphalidae: Satyrinae), simultaneously with *E.stigmatica*, based on three specimens collected by Smith in Chapada; prior to Godman (1905), Godman also described eight riodinid species based on specimens collected by Smith in Chapada (Godman 1903); Williston (1888) also incorporated at least 400 specimens representing more than 80 dipteran species sampled by Smith in Chapada when he described *M.inermis* in his “Diptera Braziliana”. Williston’s (1888) introduction begins with “More than a year ago Mr. Herbert H. Smith, who is well known to zoologists for his writings on Brazil, placed in my hand for study a collection of Diptera made by him during the past few years in Southern Brazil”. Given the mention of “more than a year ago,” as well as having the work published in 1888, implies that Williston received materials subsequent to Smith’s various excursions to Chapada and other sites he presumably visited between 1881 and 1886, perhaps in late 1886 upon his return from Brazil or at some point in 1887. The statement “made by him [H.H. Smith] during the past few years” refers to specimens originating from Smith’s entomological expeditions to Chapada and other places in Brazil which took place between 1881 and 1886, not samples obtained (if any) by Smith in the 1870’s – this is supported by the fact that a number of dipteran specimens examined and incorporated in Williston (1888) were collected in Chapada. Both Samuel W. Williston and F. D. Godman worked on Biologia Centrali-Americana: zoology, botany and archaeology, a ground-breaking work on the Central American fauna and flora, to which Smith contributed with materials from his trip to Mexico in 1888–1889 (e.g., Osten Sacken 1887). Although Godman’s (1903, 1905) aforementioned works appeared after Smith’s travel to Mexico, Williston (1888) already had Smith’s entomological samples from Brazil prior to 1888, thus it is possible that Godman also had Smith’s Brazilian Lepidoptera specimens in his hands before 1888. In order to develop this discussion further, it is important to take into account Drake’s (1922) work on the hemipteran family “Tingitidae” [= Tingidae] based predominantly on specimens housed at the Carnegie Museum of Natural History. Drake (1922: 361) referred to a single specimen as a paratype in the author’s collection from “Entre Rios, Brazil” in his description of *Amblystiranyctalis* Drake, 1922 (Hemiptera: Tingitidae). Like *E.stigmatica* and *M.inermis*, this paratype of *A.nyctalis* housed at USNM also bears a label stating that it was collected in September of an unknown year (Fig. 7D). The holotype of *A.nyctalis* is a male from Chapada. Drake (1922: 361) stated that these two specimens were “collected by Mr. and Mrs. H. H. Smith”. Herbert H. Smith married Miss Amelia Woolworth Smith on October 5, 1880 and she participated in Smith’s trip to Brazil between 1881 and 1886 (Holland 1919). Thus, the specimen from “Entre Rios, Brazil” used in Drake’s (1922) description of *A.nyctalis* is based on materials collected while Smith and his wife made their headquarters in Chapada between 1882 and 1886. Among nine species of *Amblystira* known to occur in South America, two have been recorded from Argentina, both in the province of Misiones (Montemayor 2010). Furthermore, by consulting with an expert, the locality of *A.nyctalis* is more likely to be Brazil, not in northeastern Argentina (Sara Montemayor, pers. comm., November 2022). From a similar biogeographical point of view, we can call into question the type locality of *E.stigmatica* being a site in northeastern Argentina, since records for *Caeruleuptychia* from this region are not known to us to date.

Unlike Smith’s study of the coffee industry at Entre Rios, Brazil, in the late 1870s, documented in Smith (1879a, b), we were unable to find any statement in the existing literature which can be accepted without ambiguity to support that Smith actually visited Entre Rios (= Três Rios) in Brazil between the years of 1881 and 1886. Nevertheless, some inference can be made. For example, in chapter 24 of his “Do…Cuyabá”, Smith (1922: 247) wrote “É de notar que nossas colecções paraguayas [de insectos] differiam muito das que fizeramos na região costeira do Brasil” [It is to be noted that our Paraguayan [insect] collections differed greatly from those we did in the coastal region of Brazil], which implies Smith did collect insects in the coastal region of Brazil (i.e., the Atlantic Forest). It must be noted that Chapada, located in Mato Grosso is unlikely to be considered as a site lying in the coastal region of Brazil, thus leaving the possibility of Smith collecting insects elsewhere between 1881 and 1886. De Abreu (1922) stated that in addition to staying for four years in Chapada, Smith spent a few months in Pará, ten days in Pernambuco, six months in Rio de Janeiro, and six months in Rio Grande do Sul, starting from May 1881. Thus, it is possible that Smith visited Entre Rios (= Três Rios) while residing in Rio de Janeiro for six months, especially since he would have been familiar with the region due to his prior visit in the 1870s. This possibility can also be supported based on the botanical materials collected by Smith and currently housed at the New York Botanical Garden’s C. V. Starr Virtual Herbarium (https://sweetgum.nybg.org/science/vh/). Among 17 botanical samples in this Herbarium collected by Smith, 16 samples are from Mato Grosso, whilst there exists a single specimen from Minas Gerais. Considering the fact that the whereabouts of the majority of the botanical samples amassed by Smith remain unknown (Kunzler et al. 2011), it is not clear how these materials ended up in New York. Notwithstanding this situation, the presence of an individual from Minas Gerais among samples from Mato Grosso, can be seen as evidence to support the supposition that Smith visited Entre Rios at some point between 1881 and 1886. Entre Rios (= Três Rios) is situated near the border of the states of Rio de Janeiro and Minas Gerais, and the Estrada de Ferro D. Pedro II railway would have passed through Entre Rios on its way from Rio de Janeiro to various stations in Minas Gerais. William J. Holland, a then director of the Carnegie Museum of Natural History, estimated that approximately 30,000 species and 200,000 insect specimens entered the collection of the Carnegie Museum of Natural History (Holland 1919). These figures illustrate a discrepancy between the number of insect species collected by Smith at Chapada (10,000 species) (de Abreu 1922), suggesting that he conducted collecting trips in various other places besides Chapada, including the areas around Rio de Janeiro. Indeed, Williston (1888) did cite more than 130 specimens representing 36 dipteran species collected by Smith in “Rio de Janeiro”, reinforcing Smith’s entomological survey in the Atlantic Forest between 1881 and 1886.

In the present study, we call into question the purported type locality of *E.stigmatica* representing a site in northeastern Argentina. Based on the discussion developed above, the holotype of *E.stigmatica* was more likely to have been collected in Entre Rios in the Brazilian state of Rio de Janeiro, a place currently known as Três Rios. Herbert H. Smith likely collected the holotype of *E.stigmatica* in September 1881. The argumentation supporting the conclusion regarding the type locality of *E.stigmatica* in the present study can be summarized as follows:

1. Smith visited Entre Rios (= Três Rios) in the state of Rio de Janeiro in the 1870s.
2. Given the proximity, he was able to revisit Entre Rios (= Três Rios) while residing in Rio de Janeiro for six months in 1881.
3. Simultaneous descriptions of taxa based on specimens from both Entre Rios and Chapada by Godman (1905), Williston (1888), and Drake (1922) suggested specimens from Entre Rios were also part of the entomological samples brought back to the United States by Smith in 1886.
4. Entre Rios was considered to be in Argentina by Godman, but Williston (1888) did not associate the country of origin in his description (also reflected in the type label), and Drake (1922) linked Entre Rios with Brazil, not Argentina.
5. Type materials of *E.stigmatica*, *M.inermis*, and *A.nyctalis* all bear labels indicating that they were collected in September (see Figs 2, 7C, D).
6. Evidence suggests Smith did collect insects apart from Chapada, between the years of 1881 and 1886 (see immediately preceding paragraph).
7. The paratype of *A.nyctalis* was collected after Smith’s marriage in 1880.
8. Current understanding of *Amblystira* and *Caeruleuptychia* distribution suggests Rio de Janeiro to be a more plausible locality for *A.nyctalis* and *E.stigmatica* (no records exist for these two genera from Entre Rios province in Argentina).

Finally, we note that the aforementioned conclusion of the type locality reinforces the current taxonomic status of *E.stigmatica* being a junior subjective synonym of *E.cyanites*. The type locality of *E.stigmatica* likely being Entre Rios (= Três Rios) situated in the state of Rio de Janeiro, coupled with six historical specimens from Rio de Janeiro and São Paulo housed at DZUP and MfN (see above), suggests that *C.cyanites* occurs in the southern part of the Atlantic Forest. Nevertheless, the three females from “Araras, São Paulo” housed at DZUP may be mislabeled since the senior author (AVLF) and colleagues have sampled extensively in the region (e.g., Brown and Freitas 2000) without documenting this species. Despite the lack of recent materials from this region, as well as provenance of three specimens currently unknown, two males from São Paulo housed at MfN do fall into the concept of *C.cyanites* discussed above based on the forewing length and scent patch size as shown in Fig. 1B. Also, two females from Rio de Janeiro (in DZUP and MfN) do phenotypically agree with the sequenced female from Espírito Santo (BC-DZ-007). As supported by a number of butterfly taxa (e.g., *Scadareckia* (Hübner, 1808); *Heliconiusnattereri* C. Felder & R. Felder, 1865; *Pierellahyalinus* (Gmelin, [1790]) (all Nymphalidae)), as well as literature investigating inter-specific genetic divergence (e.g., Pirani et al. 2022), Espírito Santo, northeastern Minas Gerais and the coastal region of Bahia can be characterized as faunistically different from the southern region of the Atlantic Forest, including the state of Rio de Janeiro. Apparently, no records of *Caeruleuptychia* exist in inventories compiled for butterflies from Rio de Janeiro (e.g., Capronnier 1881; Soares et al. 2011), suggesting its rarity and perhaps disjunct distribution. In the absence of genetic data from the holotype of *E.stigmatica*, one may argue that the available evidence is insufficient to draw conclusion as to its identity. On the other hand, it is apparent that there is only a single species of *Caeruleuptychia* with blue phenotype known from the Atlantic Forest. If we accept the type locality of *E.stigmatica* is in the state of Rio de Janeiro, coupled with the existence of two known male *Caeruleuptychia* from this region with the same habitus (both in MfN), it seems reasonable to apply the same species-group name *stigmatica* to these historical specimens in DZUP and MfN and regard them as conspecific with the holotype of *E.stigmatica*. Consequently, we parsimoniously apply the same specific epithet to specimens from Espírito Santo, northeastern Minas Gerais, and the coastal region of Bahia. We provided evidence from external morphology and literature supporting that *E.cyanites* is likely from southeastern Brazil as well. Thus, it seems reasonable to retain the synonymy between *E.stigmatica* and *E.cyanites*, although we admit that this taxonomic hypothesis is subject to revision if additional evidence surfaces. We thus settle the matter here by designating a lectotype for *E.cyanites* based on the male syntype discussed above and consider this species-group name to represent a species in *Caeruleuptychia* known to occur in the Atlantic Forest as a senior synonym of *E.stigmatica* (lectotype designation).

## Supplementary Material

XML Treatment for Caeruleuptychia cyanites

XML Treatment for Euptychia stigmatica

XML Treatment for Caeruleuptychia harrisi

XML Treatment for Caeruleuptychia aemulatio

## ﻿Additional information

### Author ORCIDs

Shinichi Nakahara https://orcid.org/0000-0002-2912-343X

Kaylin Kleckner https://orcid.org/0000-0002-8554-1556

Eduardo P. Barbosa https://orcid.org/0000-0002-8885-454X

Giselle M. Lourenço https://orcid.org/0000-0003-1452-641X

Mirna M. Casagrande https://orcid.org/0000-0002-6076-8463

Keith R. Willmott https://orcid.org/0000-0002-9228-0219

André V. L. Freitas https://orcid.org/0000-0002-5763-4990

### Data availability

All of the data that support the findings of this study are available in the main text or Supplementary Information.
